# Status Epilepticus and Blindness in a Patient with Carfilzomib-Associated Posterior Reversible Encephalopathy Syndrome

**DOI:** 10.7759/cureus.1041

**Published:** 2017-02-19

**Authors:** Salam Kadhem, Rawaa Ebrahem, Scott Cooper, Emily Manlove, Ricky Lee

**Affiliations:** 1 Internal Medicine, University of Kansas School of Medicine-Wichita; 2 Family Medicine, University of Kansas School of Medicine-Wichita; 3 Internal Medicine, Via Christi Hospital Wichita

**Keywords:** relapsing myeloma, status epileptics, blindness, carfilzomib, vasogenic edema

## Abstract

Posterior reversible encephalopathy syndrome (PRES) is a neurological condition characterized by headaches, visual disturbances, and seizures. A magnetic resonance imaging (MRI) scan of an affected brain typically shows symmetrical white matter edema in the posterior cerebral hemispheres. The onset of PRES can constitute a medical emergency, especially when accompanied by status epilepticus. If promptly recognized and treated, the clinical syndrome and associated radiological findings are usually resolved in a matter of weeks or months. Carfilzomib is a proteasome inhibitor that is newly approved for relapsing myeloma in a patient who has received one or more lines of therapy. In this paper, we report on a 52-year-old female on carfilzomib for multiple myeloma who developed PRES following her second dose of treatment. She was admitted for chronic obstructive pulmonary disease (COPD) exacerbation, and while she was in the hospital, she developed a severe headache, blindness, and status epilepticus. A brain MRI showed signs consistent with PRES. After carfilzomib was discontinued, her symptoms resolved within three days. Unfortunately, the patient passed away shortly after being discharged, so there was no opportunity to perform a repeat MRI.

## Introduction

Posterior reversible encephalopathy syndrome (PRES) is a neurological condition whose symptoms include headaches, visual disturbances, and seizures [1]. A magnetic resonance imaging (MRI) scan of an affected brain typically shows symmetrical white matter edema in the posterior cerebral hemispheres, but this can also present in other anatomical areas as well [1-2]. Recent developments in drug manufacturing and the increasing number of newly marketed medicines are associated with newly reported adverse events. Carfilzomib is one of the recently approved proteasome inhibitors for refractory and relapsing multiple myeloma in patients who have received one or more lines of therapy. In July 2015, the US Food and Drug Administration (FDA) approved this medication as a component of combination therapy with dexamethasone or lenalidomide [3]. There were no apparent adverse neurological events noted in the studies conducted prior to its FDA approval; however, a limited number of cases of carfilzomib-associated PRES have been reported post-marketing [4-5]. Early recognition of this condition is paramount for proper treatment and to avoid permanent neurological damage. 

Informed consent was obtained from the patient for this study.

## Case presentation

**Figure 1:**
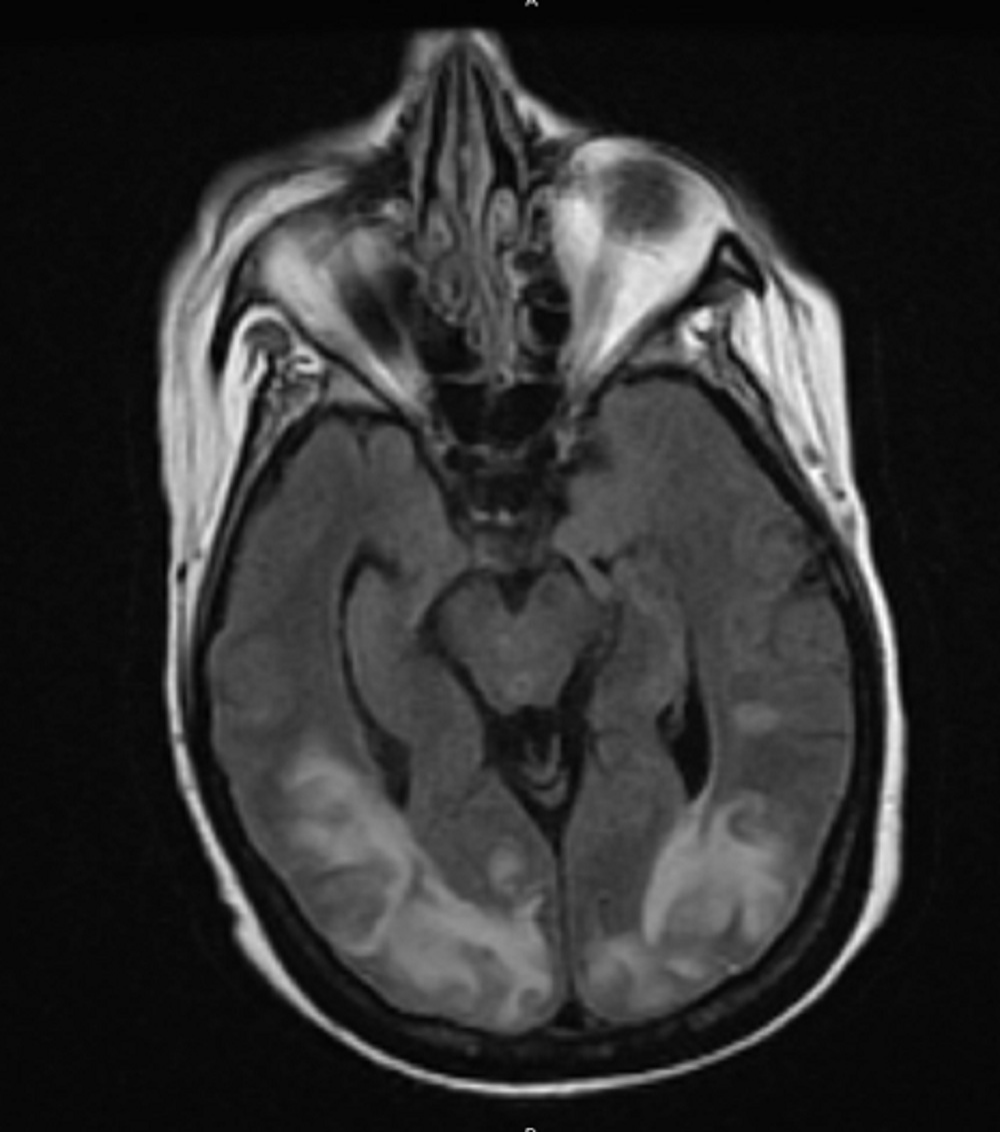
MRI brain showed T2/FLAIR signal hyperintensity over the bilateral posterior head regions, consistent with PRES

**Figure 2:**
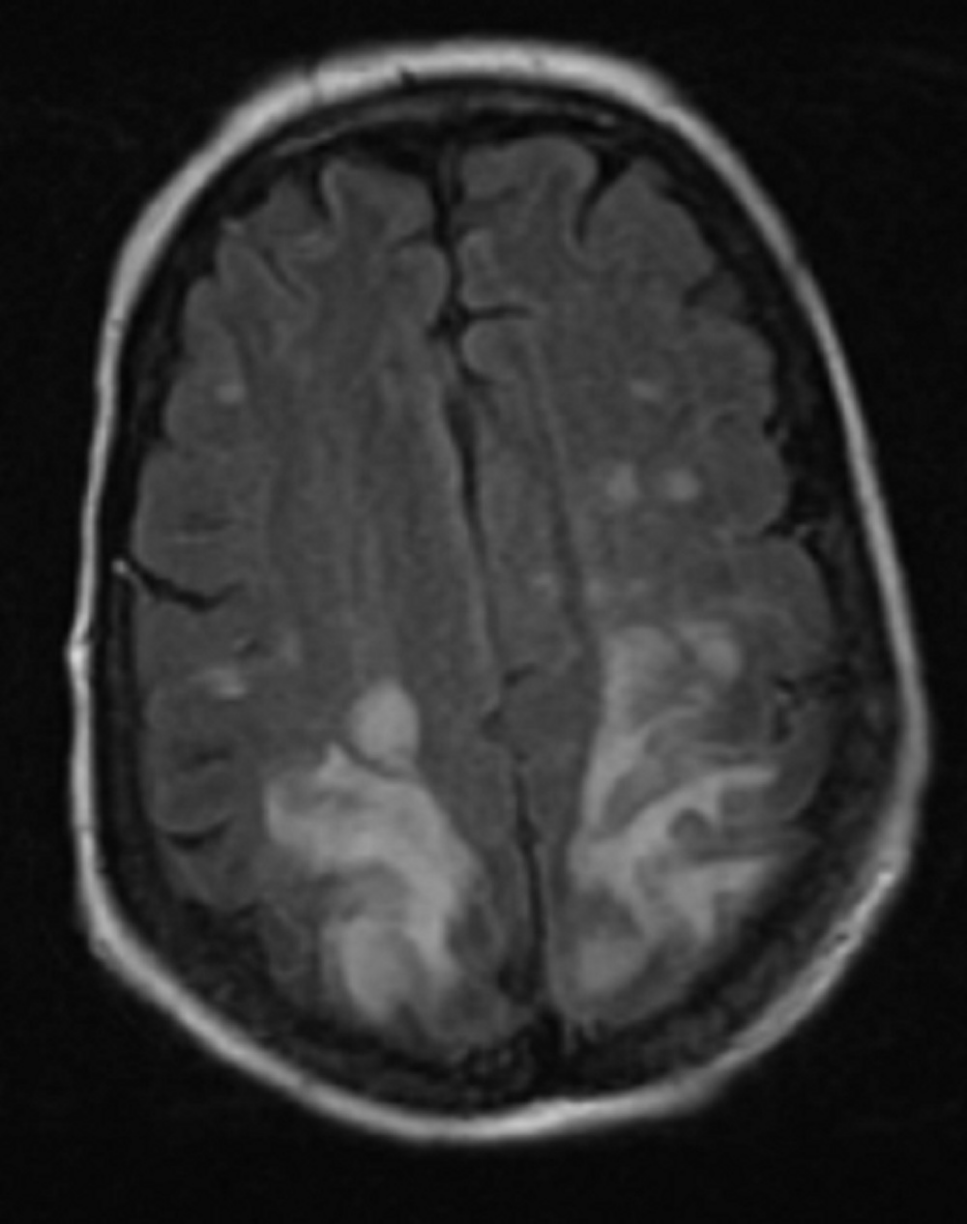
MRI brain showed T2/FLAIR signal hyperintensity over the bilateral posterior head regions, consistent with PRES

A 52-year-old female with a past medical history significant for refractory multiple myeloma was admitted to the hospital for chronic obstructive pulmonary disease (COPD) exacerbation. She received her second dose of carfilzomib seven days prior to hospitalization. On the fourth day of hospitalization, she reported a severe headache and blindness. This was followed by status epilepticus, requiring treatment with levetiracetam. An MRI of the brain showed no abnormality in the diffusion-weighted imaging (DWI) sequence that would suggest acute ischemic stroke. The MRI showed fluid-attenuated inversion recovery (FLAIR) and T2 signal hyperintensity over the bilateral parietal and occipital lobes that extended into the posterior temporal lobes. The MRI’s brain findings were consistent with PRES (Figures 1-2).

An electroencephalogram (EEG) showed maximal background slowing over the bilateral occipital regions, as well as left occipital potentially epileptogenic abnormalities.

The patient was not hypertensive recently and had not been treated with any other immunotherapy apart from the recently started carfilzomib. As no other inciting cause was identified, the patient was presumptively diagnosed with carfilzomib-associated PRES. The headache and visual deficit resolved a few days after discontinuation of carfilzomib (was not given as scheduled during her hospital stay). The oncologist recommended lenalidomide as an alternative therapy for the resistant multiple myeloma, and the patient was discharged home on levetiracetam for seizure prophylaxis. Unfortunately, the patient died two months later due to multiple comorbidities. For this reason, a follow-up MRI brain scan, which is typically performed three months after diagnosis, was not available for comparison.

## Discussion

PRES is an acute neurological syndrome characterized by seizures, encephalopathy, headaches, and visual disturbances [1]. These symptoms develop acutely over a few hours and can have a wide range of manifestations, depending on the area of the brain involved. Radiological findings show areas of vasogenic edema due to disruption of the blood-brain barrier’s integrity [1,6]. Imaging abnormalities are typically seen in the parietooccipital lobe, posterior frontal lobes, and inferior temporal lobes [6]. Risk factors for PRES include renal failure, hypertension, vascular disease, autoimmune disorders, and exposure to immunosuppressive medications. Although the mechanism by which proteasome inhibitor immunosuppressive medications (such as carfilzomib) influence the development of PRES is yet to be fully understood, it has been hypothesized that decreased transcription of growth factors, including vascular endothelial growth factor (VEGF), eventually leads to endothelial dysfunction, vasospasm, and blood-brain barrier disruption [5]. Prior to this report, there have only been very few cases of carfilzomib-related PRES. Our case illustrates the possibility of status epilepticus in patients receiving carfilzomib treatment, which is a medical emergency. If promptly recognized and treated, the clinical syndrome and radiological findings usually resolve within a matter of weeks to months, without the residual deficit.

## Conclusions

Treatment for clinical and radiological diagnosis of PRES includes close neurologic monitoring, addressing potential provoking factors, and supportive care. Given the high likelihood of full recovery after cessation of the causative agent, clinicians should be aware of the potential association between carfilzomib and PRES. With such clinical presentations, a thorough history, physical review, medication review, and early brain MRI should be conducted to confirm the diagnosis and rule out other serious diagnoses.
